# Importance of plasticity and local adaptation for coping with changing salinity in coastal areas: a test case with barnacles in the Baltic Sea

**DOI:** 10.1186/1471-2148-14-156

**Published:** 2014-07-19

**Authors:** Anna-Lisa Wrange, Carl André, Torbjörn Lundh, Ulrika Lind, Anders Blomberg, Per J Jonsson, Jon N Havenhand

**Affiliations:** 1 Department of Biological and Environmental Sciences-Tjärnö, University of Gothenburg, S-45296 Strömstad, Sweden; 2 Mathematical Sciences, Chalmers University of Technology and Mathematical Sciences, University of Gothenburg, Gothenburg, Sweden; 3 Department of Chemistry and Molecular Biology, University of Gothenburg, Gothenburg, Sweden

**Keywords:** Evolutionary change, Phenotypic plasticity, Baltic Sea, Crustacea, Common-garden experiment, *Balanus (Amphibalanus) improvisus*, Gompertz growth model

## Abstract

**Background:**

Salinity plays an important role in shaping coastal marine communities. Near-future climate predictions indicate that salinity will decrease in many shallow coastal areas due to increased precipitation; however, few studies have addressed this issue. The ability of ecosystems to cope with future changes will depend on species’ capacities to acclimatise or adapt to new environmental conditions. Here, we investigated the effects of a strong salinity gradient (the Baltic Sea system – Baltic, Kattegat, Skagerrak) on plasticity and adaptations in the euryhaline barnacle *Balanus improvisus.* We used a common-garden approach, where multiple batches of newly settled barnacles from each of three different geographical areas along the Skagerrak-Baltic salinity gradient were exposed to corresponding native salinities (6, 15 and 30 PSU), and phenotypic traits including mortality, growth, shell strength, condition index and reproductive maturity were recorded.

**Results:**

We found that *B. improvisus* was highly euryhaline, but had highest growth and reproductive maturity at intermediate salinities. We also found that low salinity had negative effects on other fitness-related traits including initial growth and shell strength, although mortality was also lowest in low salinity. Overall, differences between populations in most measured traits were weak, indicating little local adaptation to salinity. Nonetheless, we observed some population-specific responses – notably that populations from high salinity grew stronger shells in their native salinity compared to the other populations, possibly indicating adaptation to differences in local predation pressure.

**Conclusions:**

Our study shows that *B. improvisus* is an example of a true brackish-water species, and that plastic responses are more likely than evolutionary tracking in coping with future changes in coastal salinity.

## Background

Future climate-driven changes in the marine environment are projected to include decreased salinity in many coastal areas due to increased precipitation and enhanced freshwater run-off [1-3]. Most climate change research to date has focused on effects of increasing temperatures and ocean acidification, rather than salinity [4-6]. Salinity plays an important role in shaping the distribution of marine species and future alterations in salinity may pose major ecological challenges to organisms inhabiting coastal areas [7]. Changes in salinity are known to have direct or indirect effects on survival, metabolism, growth, reproduction, and/or osmotic balance in aquatic organisms [8-12]. Future salinity shifts in coastal areas may therefore impose strong selection on species inhabiting these areas, leading to changes in species composition and the evolution of new adaptations. Understanding how marine organisms respond to environmental changes and how rapidly new adaptations can evolve is key for predicting how ecosystems will respond to global environmental change in the future.

**Figure 1:**
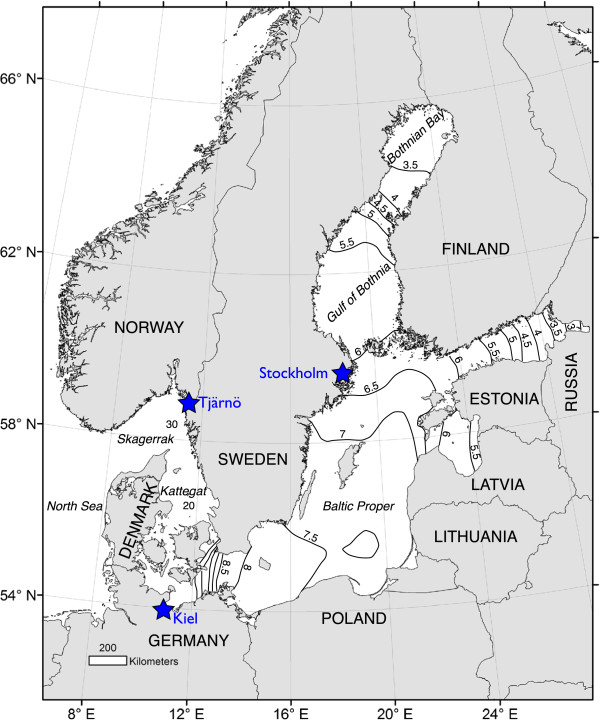
**Map of the study area.** The Skagerrak-Baltic Sea region is characterized by a strong salinity gradient. The sites where the barnacles were collected (shown as stars) include: Stockholm (59.286 E; 18.708 N), Kiel fjord (54.327 E; 10.185 N) and Tjärnö (58.881E; 11.134 N).

In the Baltic Sea, salinity forms a strong environmental gradient that determines the composition and distribution of species [13,14]. It is one of the world’s largest semi-enclosed brackish seas and reached its present brackish state about 8000 years ago [15,16]. Since then, the Baltic Sea has been colonised by organisms from both freshwater and marine environments [16,17]. The sea surface salinity along this gradient ranges from < 3 PSU (practical salinity units) in the northern Bothnian Bay (Figure 1) to approximately 30 PSU at the border to the North Sea [13]. Limited water exchange with surrounding seas and almost no tidal flow make salinity conditions in the Baltic Sea relatively stable compared to many other coastal areas [18,19]. Species diversity and within-species genetic diversity are markedly lower inside the brackish Baltic Sea compared to adjacent areas, which to a large extent is attributed to these marginal environmental conditions creating specific selection pressures and promoting the evolution of local adaptations [20-22]. Recent modelling of future climate scenarios in the Baltic Sea indicates that increased precipitation will lead to reduced salinity [3,23], which could potentially result in dramatic shifts in species’ distributions. Such shifts would also likely influence ecological interactions and ecosystem functions.

Organisms can respond to changes in salinity through plastic responses involving molecular (e.g. gene expression), physiological, morphological, behavioural or life history trait changes, without genotypic changes occurring through selection [24]. Here, we mainly consider phenotype plasticity in the context of “phenotypic buffering”, i.e. a plasticity that maintains a functional phenotype despite external disturbances [25]. From an evolutionary perspective, it has been suggested that phenotypic plasticity can act as a buffer against selection that may delay or even preclude the evolution of local adaptations. However, it can also be considered a source of novel opportunities upon which selection can act, e.g. by facilitating the transition to new fitness levels while maintaining a large population size [26-28]. In either case, phenotypic plasticity is likely to play an important role in the ability of species to tolerate future environmental changes [29]. Being highly plastic is often assumed to involve a cost, which would explain why plasticity is not universal [30,31]. Consequently, it has been suggested that in more stable environments, where (costly) high plasticity is not required, adaptations to a narrower range of environmental conditions may be selected for, resulting in a loss of phenotypic plasticity [32-34]. Adaptation, in contrast to phenotypic plasticity, always involves a genetic change, resulting from selection acting on fitness-related phenotypic traits. Although there is mounting evidence that local adaptation may be common in the marine environment, there are few studies on the relative role of evolutionary responses versus plasticity to changing salinity in marine invertebrates [35], but see [36].

Barnacles (Crustacea; Cirripeda) generally display high tolerance to fluctuating environments. They make up an important component of intertidal and estuarine communities, where they are frequently exposed to variation in salinity, as well as fluctuating temperature and desiccation [37]. The bay barnacle *Balanus (Amphibalanus) improvisus*[38] is the only barnacle species recorded from the Baltic Sea, but it is also found in other coastal areas and estuaries worldwide [39]. *B. improvisus* is believed to originate from the eastern North America and was first recorded in the Baltic Sea in the mid 1800’s [40]. Compared to most other barnacle species, *B. improvisus* can tolerate a very wide range of salinities, from <1 to 35 PSU, however, salinities below 3 PSU have been reported to have long-term negative impacts on fitness [41-43]. The molecular and cellular mechanisms behind this broad salinity tolerance are poorly known, although it has been proposed that *B. improvisus* can alternate between being a passive osmoconformer in high salinities (>15 PSU) and actively osmoregulate in salinities below 15 PSU [41]. It was recently shown that alternatively spliced variants of Na^+^/K^+^ ATPases were differentially expressed in response to reduced salinity giving further support for plasticity through active osmoregulation [44].

If there is indeed a cost to the euryhalinity (maintaining high physiological plasticity in relation to salinity) of *B. improvisus*, then this capacity may be reduced, or even lost, in populations living in a stable and low salinity environment such as the Baltic Sea. Previous studies comparing the effects of salinity in different barnacle species found that *B. improvisus* had a broad tolerance and fed actively in most salinity conditions, and that the response depended on previous salinity history [45]. More specifically, *B. improvisus* from the Baltic Sea showed highest activity in low salinities (6 PSU), compared to populations of *B. improvisus* from England (higher salinities), potentially indicating local adaptation to the Baltic environment [45]. This may indicate that rapid evolution had occurred in Baltic Sea populations of *B. improvisus* over a timescale of a few hundred years after colonisation. However, the study by Davenport [45] only tested short-term exposures to different salinities without appropriate acclimation of barnacles collected from the field, which limits the ability to draw conclusions about the degree of local adaptation. By performing long-term controlled experiments, starting from early post-settlement and following barnacles until sexual maturity, we can better understand how salinity affects a wide range of phenotypic traits and reveal which traits may be more exposed to selection in different salinity conditions, potentially resulting in local adaptations.

Here, we investigated whether the broad distribution of *B. improvisus* along the strong salinity gradient of the Baltic Sea can be explained by physiological plasticity or if local adaptations to various salinity regimes have evolved in populations along the gradient. We used a common-garden experiment in which multiple barnacle populations sampled along the salinity gradient were grown from immediate post-settlement to reproductive maturity under different salinity treatments. We measured several fitness-related traits including growth, mortality, shell strength and reproductive maturity.

## Methods

### Sampling of brood stock barnacles and larval culture

Several hundred adult barnacles (*Balanus (Amphibalanus) improvisus*) were collected on settling panels from three different populations in: i) the Stockholm archipelago, Baltic Sea (Sweden, 4-6 PSU [19]; 59.286 E; 18.708 N), ii) the Kiel Fjord, Baltic Sea (Germany, 14-17 PSU [46]; 54.327 E; 10.185 N) and iii) Tjärnö, Swedish west coast (Sweden, 22-30 PSU [47]; 58.881E; 11.134 N) during July to August 2011 (Figure 1). Settled barnacles were transported to the laboratory at Tjärnö (Sven Lovén Centre for Marine Sciences) and kept in large re-circulating systems at their native mean salinities (6, 15 and 30 PSU) and at a constant temperature of 19°C. Adult barnacles were fed daily with newly hatched *Artemia* sp*. ad libitum*. As in other barnacle species, *B. improvisus* produces free-swimming larvae (six nauplius stages followed by a non-feeding cyprid stage) with pelagic dispersal for up to several weeks before they settle on hard substrates and metamorphose into the adult stage [48]. Nauplius larvae were obtained through natural spawning of the adults in a modified laboratory culture system described previously [49]. Nauplii were cultured (at maximum 500 nauplii L^-1^) until the cyprid stage in their native salinities, which were created by mixing filtered seawater (0.2 μm) with filtered tap water (0.2 μm) and fed with a mixture of the microalgae *Thalassiosira pseudonana* and *Skeletonema marinoi* (at a ratio of 40:60, respectively, and in *ad libitum*). These microalgae were also cultured at different salinities (10 PSU, 15 PSU and 30 PSU) in order to reduce osmotic disturbances when transferring algae to the larval cultures. We took into account the possible natural variability between larval batches by collecting larvae from the adult barnacle cultures (consisting of several hundred individuals each) on four different occasions during two weeks in September-October 2011, representing four replicate batches of larvae (presumably with different parents, and thus different genotypes). Larval batches were used as level of replication for each population, since natural variability in larval performance between batches has been observed previously [50]. Cyprid larvae from each batch were settled on acrylic plastic (*Plexiglas*) panels and were transferred to the experimental system (see below) after eight days, leaving enough time for the larvae to attach and metamorphose before the start of the experiment.

### Experimental setup

The experiment involved three salinity treatments (6, 15 and 30 PSU), corresponding to the native mean salinities for the three barnacle populations studied (Stockholm, Kiel and Tjärnö, respectively). From each of the four batches obtained per population, two panels with newly settled barnacles (eight days old) were placed in separate aquaria in each of the three salinity treatments, resulting in a split-plot design [51]. Three large independent re-circulating systems (370 l each) were used, each consisting of 24 aquaria (6 l each). The flow through each aquarium was 20-25 l/h. Each system had a biological filter (Fluval filter 405) with coral rubble, activated charcoal and nitrifying bacteria added to maintain water quality and avoid build-up of nitrogenous waste products. In addition, water in each system was completely replaced every two weeks, at which time the culture systems were cleaned, drained and treatment combinations were moved to a new system within the same room to avoid confounding the effects of salinity with effects of aquarium location within the room. The experiment was maintained at 20°C and a light regime of 14:10 h (L:D). The different salinities were obtained by mixing filtered deep saltwater (0.2 μm) from the Kosterfjord (30-34 PSU, total alkalinity, A_T_, of 2186-2290 μmol l^-1^) with filtered tap water (A_T_ of 369 μmol l^-1^). Water quality variables including temperature, salinity, A_T_, NH_3_ and NH_4_ were monitored routinely during the experiment (Additional file 1: Table S1). Salinity was adjusted by adding freshwater whenever needed. Barnacles were fed a mixture of microalgae (*Skeletonema marinoi* and *Chaetoceros calcitrans*) at ~ 20,000 cells ml^-1^ and 30,000 cells ml^-1^, respectively. Algal cell concentrations were checked regularly using a Multisizer™ 3 Coulter Counter (Beckman Coulter) and levels were adjusted to maintain stable concentrations *ad libitum* throughout the experiment. The algal cell concentration in the systems never reached below 5,000 cells ml^-1^. Chlorophyll content of the algal cultures and experimental systems were also checked for possible degradation of food quality, assessed by a spectrophotometric trichromatic method [52], which showed no signs of degradation. After four weeks, the barnacle diet was complemented with newly hatched *Artemia* (ca. 3,000 *Artemia* per aquarium) added every second day. Barnacles were cultured in the experimental systems for a total of nine weeks.

### Growth modelling and condition index

**Figure 2:**
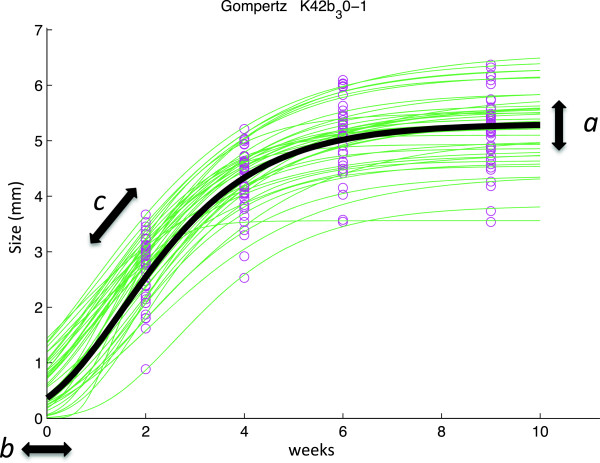
**Illustration of the Gompertz growth model.** The Gompertz growth model is a sigmoid function described by three parameters; *a* (asymptote), *b* (lag phase) and *c* (maximal growth rate). Model parameters for each replicate panel with barnacles, were obtained by first fitting a model (thin lines) to shell diameter data (circles) for each barnacle on a panel (measurements at four different times) and then calculating an average model from these fittings for each replicate panel (thick line).

Barnacle growth was recorded photographically every two weeks, (Olympus E5 DSLR, 50 mm F1.8 Macro lens). To avoid overcrowding, excess barnacles were removed from panels during the first two weeks, and subsequently whenever barnacles came in contact with each other. At the end of the experiment 5 - 96 barnacles remained on each panel (mean 43 ± 15 (SD)). The different barnacle densities were evenly distributed between populations and salinity treatments. Digital images were converted to binary files using ImageJ (version 1.43). An image analysis script in MATLAB (R2012a) was developed to track and measure maximum basal plate diameter (rostro-carinal) for each barnacle at four time points (after 2, 4, 6, and 9 weeks). Only barnacles that remained alive at the end of the experiment were included in the analysis. Two different growth models were fitted to the size data for each individual barnacle. The first model was the von Bertalanffy growth equation [53]:

(1) $$Y\left(t\right)=S_{\text{max}}\;\left(1-be^{-\mathrm{kt}}\right)$$

where *Y*(*t*) is the shell diameter at time *t*, *S*_*max*_ is the asymptote of the curve, *b* is the lag phase (initial growth), and *k* is the growth rate. The second model was the Gompertz growth equation [54]:

(2) $$Y\left(t\right)=ae^{be^{\mathrm{ct}}}$$

where *Y*(*t*) is the shell diameter at time *t*, *a* is the maximum shell diameter (asymptote), *b* is the lag phase or early growth phase and *c* is the maximum growth rate (how quickly the individual reaches the asymptote; note b and c are negative numbers) (Figure 2). The von Bertalanffy growth model is widely used, especially in fisheries studies, but it has also earlier been fitted to barnacle growth data [55]. The Gompertz growth model is commonly used to describe growth in fish [56], but has also been used in studies on marine invertebrates [57]. The von Bertalanffy model assumes that growth rate declines over time, whereas the Gompertz model includes assumptions of slower growth in the beginning and at the end of the growth phase (sigmoid curve). It has been suggested that a sigmoid curve (such as the Gompertz model) is more applicable to data for larval and early juvenile stages [58]. Since barnacle larvae go through a metamorphosis to become adults, including the formation of a calcareous shell, this could potentially result in reduced initial growth rate. We fitted both growth models to our data and compared them using the coefficient of determination, *R*^2^, and root mean square error (rmse). We found no statistically significant difference in fit between the two models (*t* = 0.23, *df* = 90, *P* = 0.82) based on rmse, although the Gompertz growth equation provided a better fit than von Bertalanffy in a majority of cases (47/68). We therefore used Gompertz curves in all further growth analysis. Estimates of the three Gompertz model parameters were obtained for each individual barnacle (Figure 2) and the mean value for all barnacles on each replicate panel was used in subsequent statistical analysis. Dry weight (DW) of barnacles from each batch and treatment combination was determined for 20 haphazardly selected individuals from each panel. Samples were dried (80°C, 48 h) and weighed to the nearest 0.0001 g (Sartorius CP analytical balance). Dried samples were then burned (20 h, 500°C) and the remaining inorganic material was weighed (ash weight, AW), and ash-free dry weight (AFDW) calculated. We calculated a condition index (CI) defined as the body-mass to shell-mass ratio (AFDW/AW). This measure has previously been used to evaluate physiological stress under environmental changes in invertebrates, e.g. [59,60]. Results are presented as norms of reaction, a common way to describe the relation between the genetic background (different populations) and the phenotypic responses (growth parameters) across a range of environments, e.g. different salinities [61].

### Shell strength

To investigate whether salinity affected shell strength, the strength of 20 haphazardly chosen barnacles on each panel was determined using a TAXT2i Texture Analyzer (Stable Micro Systems, 25-1 measuring cell). The pressure (compressive force) required to break the barnacle shell from above (“breaking stress”) was determined according to [59]. Maximum “breaking stress” was measured using a cylinder of 2 mm diameter pushing onto the rostral plate with a speed of 1.0 mm s^-1^. Measured shell strength (in MPa) was further normalised by dividing by the maximum shell diameter of each individual barnacle.

### Reproductive maturity

Twenty barnacles from each batch were inspected under a dissection microscope to determine their state of reproductive maturity. Gonad maturity and reproductive status could easily be determined since *B. improvisus* broods its offspring for up to several weeks before releasing them into the water column. The proportion of individuals with well-developed gonads (ovaries and/or testes – *B. improvisus* is an hermaphrodite) or fertilized eggs was recorded at the end of the experiment.

### Mortality

Mortality was estimated by comparing the number of living barnacles on each panel after two weeks with the number alive after nine weeks. Mortality estimates for the first two weeks of the experiment could not be obtained due to missing data (missing photographs), although no major mortality events were observed during this period. Mortality estimates were corrected for removal (culling) of barnacles during the experiment.

### Statistical analyses

General and generalized linear models (“aov” and “glm” respectively in R) were fitted using R-Studio (version 096.331) and SPSS (version 21). Effects of salinity and population origin on growth, shell strength, DW, AW, AFDW, CI and mortality were tested using a 2-factor ANOVA, with ‘salinity’ and ‘population’ as fixed factors. Batch was used as the level of replication in all analyses. For all the response variables we evaluated the main effects of salinity, population, and their interaction (an indicator of local adaptation). Significant interactions between salinity and population would indicate that populations respond differently to different salinity environments, which could be a result of local adaptations to a specific salinity regime. Assumptions of normality and homogeneity of variances were checked using Q/Q-plots, box plots and Levene’s test. AW was log-transformed and per cent mortality was arcsin-transformed prior to analysis. Tukey’s HSD test was used *post-hoc* to further resolve significant differences between means. Due to high frequencies of zero count data, fecundity (measured as the proportion of barnacles with fertilized eggs or mature gonads) was analysed using generalized linear models (with a Poisson distribution in R) with salinity and population as the main factors in the models. Significance of these factors was evaluated using log likelihood tests of full versus reduced models.

To test the effects of salinity and population on the overall fitness of barnacles (based on all phenotypic traits measured in this study), PERMANOVA (two-factorial, orthogonal permutational MANOVA) was applied, using Euclidian distance matrices with 9999 permutations. We also ran PERMANOVA pair-wise *post-hoc* tests for multiple comparisons. Possible trade-offs between individual phenotypic traits in different salinities were visualized and evaluated using a canonical analysis of principal coordinates (CAP) [62,63]. This type of analysis differs from unconstrained ordinations (e.g. MDS and PCA), because it identifies axes that maximize differences among groups rather than maximizing the variance explained, and thus allows a clearer focus on explicit hypotheses about e.g. salinity effects [63,64].

### Ethics statement

This work has been conducted according to relevant national and international guidelines for ethics and animal welfare, which do not include any specific requirement for barnacles.

## Results

For all the response variables we investigated the effects of salinity and population, as well as their interaction (an indicator of local adaptation). None of the interactions were statistically significant, with the exception of shell strength (see below) (Additional file 1: Table S3, S4 and S5).

### Growth

**Figure 3:**
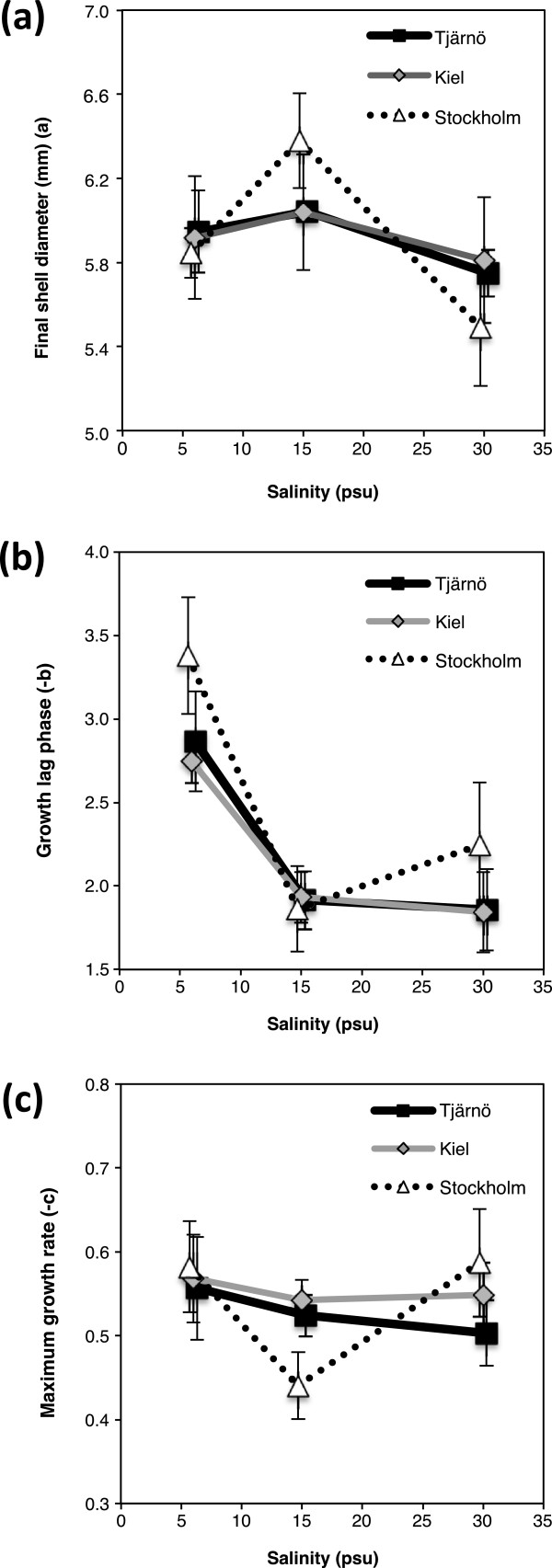
**Effects of salinity on growth.** Effects of salinity on growth in barnacles from three different populations (Stockholm, Kiel and Tjärnö), described by the growth model parameters (***a***, ***b*** and ***c***, labelled respectively) obtained from the Gompertz growth model fitted to barnacle shell diameter data (± standard error (SE), n = 4). Parameter ***a*** estimates the maximum shell diameter of the barnacles, ***b*** estimates the lag phase during early growth and ***c*** estimates the maximal growth rate, i.e. how rapidly barnacles approach the asymptote.

Salinity had a small but statistically significant effect on the size of barnacles (maximum shell diameter, as described by parameter *a* in the growth model; Figure 2) from all three tested populations (F = 3.362, *P* = 0.0497; Additional file 1: Table S3 and Figure 3a). Barnacles growing in 15 PSU had on average 8% larger shells than those in 30 PSU (Tukey’s HSD: *P* = 0.039), but were not significantly different from those in 6 PSU (Tukey’s HSD: *P* = 0.360; Figure 3a). The length of the early lag phase in growth (parameter *b* in growth model; Figure 2) was clearly influenced by salinity, where the start of growth of barnacles in 6 PSU was significantly delayed compared to the two higher salinities (15 and 30 PSU; F = 16.554, *P* < 0.001; Additional file 1: Table S3 and Figure 3b). There were no significant differences in maximum growth rate (parameter *c*; Figure 2) between salinities or between populations (Additional file 1: Table S3 and Figure 3c). Although sample sizes were small (n = 4 panels), the fitting of growth curves was good with average *r*^2^ = 0.994 ± 0.001 (SE) (Figure 2). The advantage of fitting a growth model described by several parameters, instead of simply analysing one endpoint, is that it provides a more detailed understanding of how salinity affects complex and dynamic growth patterns.

**Figure 4:**
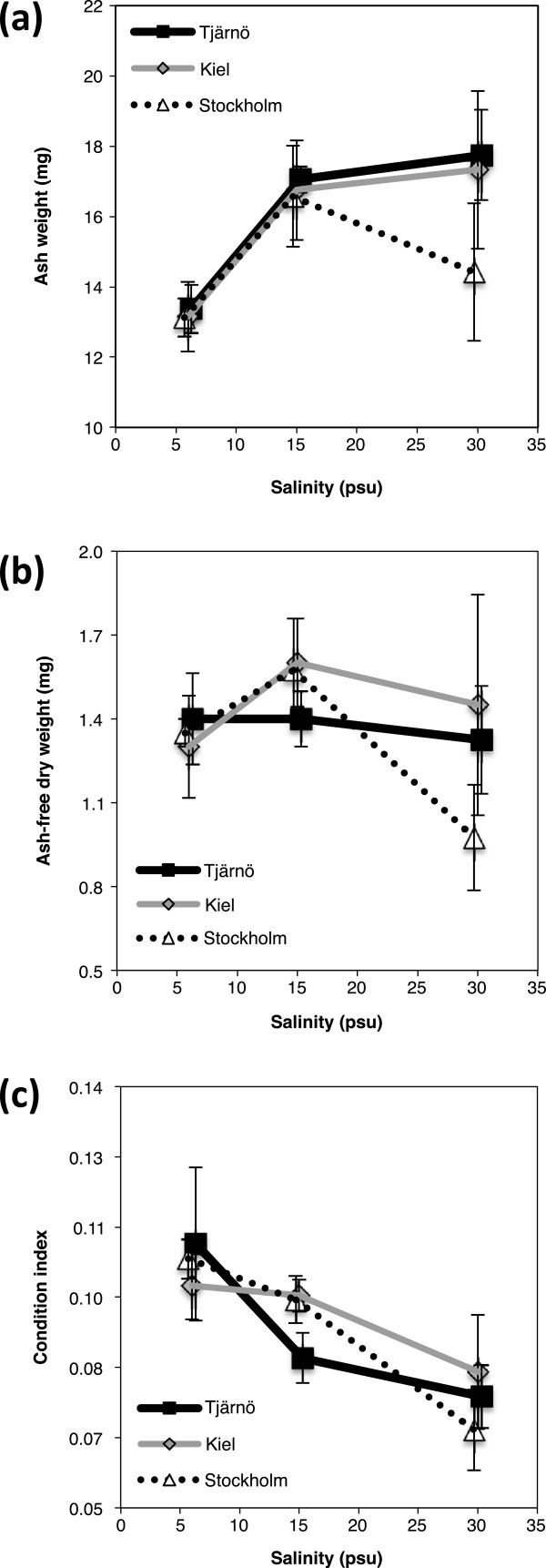
**Effects of salinity on ash weight, ash-free dry weight and condition index.** Effects of salinity on **a)** ash weight, **b)** ash-free dry weight, **c)** condition index (ratio between ash-free dry weight and dry weight) in barnacles from three different populations (Stockholm, Kiel and Tjärnö) that were grown in different salinity treatments (mean ± SE, n = 4).

Ash weight (AW) was significantly influenced by salinity (F = 6.463, *P* < 0.005; Additional file 1: Table S4), where barnacles in 6 PSU had lower AW after 9 weeks, compared to barnacles in the higher salinities (15 and 30 PSU; Figure 4a). We found no statistically significant difference in AW between populations, although in 30 PSU barnacles from the Stockholm population had lower AW than the other populations (Tjärnö and Kiel; Figure 4a). AFDW did not differ significantly between salinities or populations (Additional file 1: Table S4 and Figure 4b).

### Condition index and shell strength

**Figure 5:**
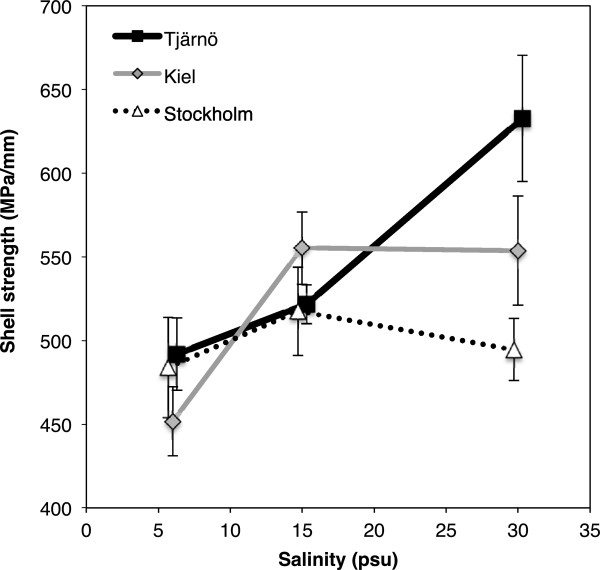
**Effects of salinity on shell strength.** Effects of salinity on shell strength in barnacles from different populations (Stockholm, Kiel and Tjärnö) that were grown in different salinity treatments (mean ± SE, n = 4). Shell strength was standardised by dividing the force required to break the shell (in MPa) by the maximum shell diameter of each barnacles.

The highest body-mass to shell-mass ratio (CI) was found in barnacles in low salinity (6 PSU), and CI decreased with increasing salinity (F = 8.658, *P* = 0.001; Additional file 1: Table S4 and Figure 4c). There were no differences in CI between populations (Additional file 1: Table S4). Furthermore, low salinity (6 PSU) resulted in weaker shells compared to higher salinities (15 and 30 PSU; F = 8.432, *P* = 0.001; Additional file 1: Table S4 and Figure 5). Importantly, however, there was a significant interaction between the effects of salinity and population on shell strength (F = 2.876, *P* = 0.042; Additional file 1: Table S4 and Figure 5). Barnacles from the high salinity environment (Tjärnö) had stronger shells in their native salinity (30 PSU) compared to the other two populations (Tukey’s HSD: *P* = 0.042; Figure 5). This positive relationship between shell strength and native salinity was not observed for the Kiel and Stockholm populations (Figure 5).

### Reproductive maturity

**Figure 6:**
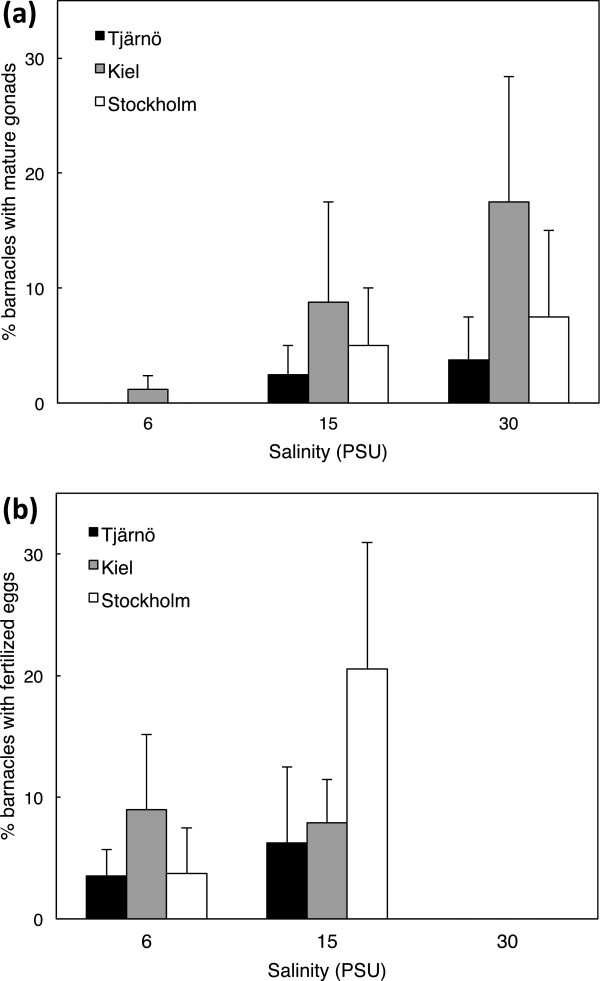
**Effects of salinity on reproduction.** Effects of salinity on reproduction in barnacles from three different populations (Stockholm, Kiel and Tjärnö). **a)** Percentage of barnacles with mature, but not yet fertilized, eggs (mean ± SE, n = 4); **b)** Percentage of barnacles with fertilized eggs (mean ± SE, n = 4).

Barnacles with mature gonads were observed in all three salinity treatments and in all three populations, but not in all combinations (Figure 6). Mature gonads were mostly observed in barnacles growing at intermediate and high salinities (15 and 30 PSU; Figure 6a). Barnacles brooding fertilized eggs, however, were only observed in the two lower salinities (6 and 15 PSU; Figure 6b). Generalized linear modelling revealed that mainly salinity but also to some extent population (but not their interaction) significantly influenced reproductive status (presence of fertilized eggs or mature gonads) (Additional file 1: Table S2). The analyses showed that the number of barnacles with fertilized eggs was best explained by a model including only salinity (P < 0.0001; Additional file 1: Table S2). This result was strongly driven by the absence of individuals with fertilized eggs in 30 PSU, (Figure 6b). For barnacles with mature gonads, but not carrying fertilized eggs, a model containing salinity and population gave the best fit (P < 0.003; Additional file 1: Table S2). For all populations, the greatest numbers of barnacles with mature gonads were observed in 30 PSU, followed by 15 PSU and 6 PSU. Barnacles from Kiel generally had the highest proportion of individuals with mature gonads in all salinities.

### Mortality

**Figure 7:**
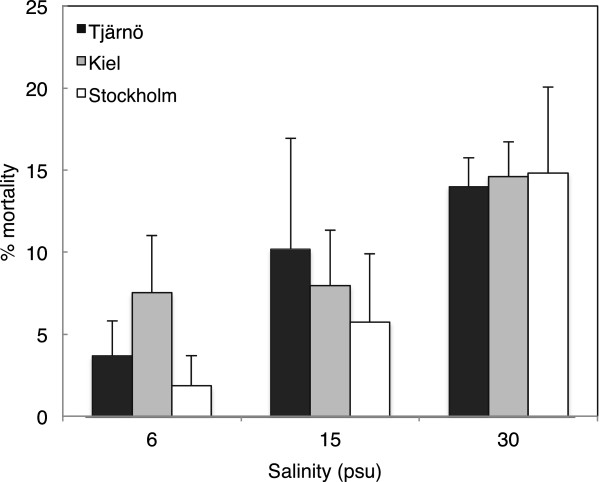
**Effects of salinity on mortality.** Effects of salinity on mortality (week 2 to week 9) in barnacles from three different populations (Stockholm, Kiel and Tjärnö) that were grown in different salinity treatments (mean ± SE; n = 2–4).

Mortality was low in all treatments and populations, ranging from 2 to 15% (Figure 7). Mortality was significantly higher in 30 PSU than in 6 PSU (F = 5.597, *P* = 0.013; Additional file 1: Table S4), however, we detected no population-specific differences in mortality (Additional file 1: Table S4).

### Trade-offs between phenotypic traits in response to salinity

**Figure 8:**
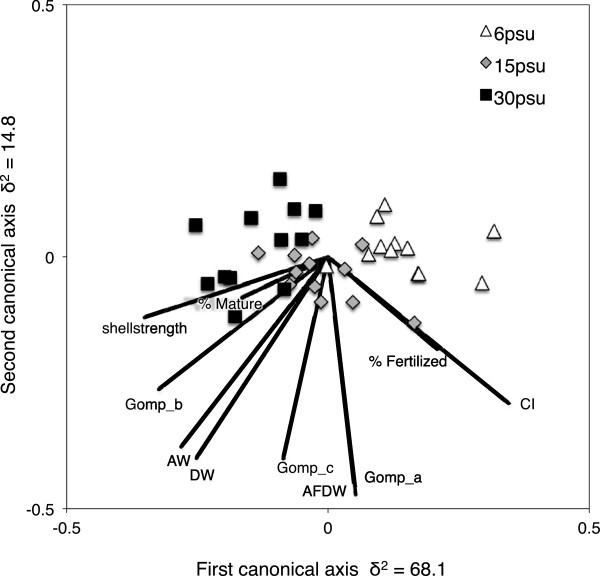
**Trade-offs between phenotypic traits in response to salinity.** Bi-plot from canonical analysis of principal coordinates (CAP) on barnacles from three populations, exposed to different salinity treatments (grouped by salinity). The first canonical axis explained 68% of the variation whereas the second axis only explained 15%. Vectors (lines) indicate the influence of different phenotypic traits in explaining the differences in responses between salinities (correlations are scaled by multiplying the original value by 0.5 to fit plot size). The phenotypic traits included are: Gomp_a, Gomp_b, Gomp_c (Gompertz growth parameters; see methods), AW (=ash weight), DW (=dry weight), AFDW (=ash-free dry weight), CI (=condition index), shell strength, % Mature and % Fertilized (=proportion of barnacles with mature gonads vs. fertilized eggs).

The CAP analysis that we used to explore effects of salinity and population on trade-offs between phenotypic traits revealed differences between salinity treatments, but not among populations (Figure 8). The first and the most important axis (δ^2^ = 68.1) separated the barnacles into three groups, based on salinity. This analysis confirmed that the traits that contributed most to this pattern were related mainly to shell strength and reproduction, although these were not 180 degrees opposed to each other as would be expected if the trade-off was complete. In high salinity (30 PSU), barnacles generally had stronger shells but delayed reproduction, whereas in low salinity (6 PSU), barnacles had weaker shells, but more fertilized eggs. In contrast, maximum size of barnacles (parameter *a* in the growth model) and AFDW did not contribute to the observed effects of salinity.

## Discussion

Our study shows that *B. improvisus* can tolerate a broad range of salinities and thus displays high phenotypic plasticity throughout its post-settlement life. Nonetheless, long-term exposure to different salinities had significant effects on fitness-related phenotypic traits such as shell-strength and fecundity. We also found evidence for a possible trade-off between traits (e.g. shell strength and reproduction), which may have important consequences in coping with changing environmental conditions. Overall responses to different salinities were similar in all populations, although differences in shell strength between populations in high salinity indicate possible local adaptation related to differences in natural predation pressure between the Baltic Sea and North Sea.

The broad salinity tolerance of *B. improvisus* was first observed by Darwin who found the species living in almost freshwater conditions in the La Plata River (Uruguay) during his voyage with the Beagle in 1831 [38]. Since then, several studies have documented the euryhaline abilities of *B. improvisus*[41,43,45,65]. Few other barnacle species show similar tolerance to low salinities [66,67], however most studies have investigated effects of short-term exposure, focusing on behavioural responses in adults, e.g. [45] or larval development, e.g. [42]. Few studies have investigated long-term effects of salinity on the post-larval life stages of barnacles [11,68]. Our study therefore contributes important new knowledge about long-term fitness-related effects of salinity exposure during the juvenile post-settlement stage until reproductive maturity.

Despite better performance in low and intermediate salinities in terms of e.g. maximum size and survival (Figures 3a and 7), our experiments revealed that long-term exposure to low salinity had negative effects on other fitness-related traits in *B. improvisus*. Growth during the first weeks after metamorphosis is recognized as a vital phase of a barnacle’s life [69], and therefore our observation of delayed onset of growth in barnacles at 6 PSU (Figure 3b) may have important consequences. Turpayeva and Simkina [43] observed a similar delay in early growth in response to low salinity in *B. improvisus*. Fitness advantages of rapid initiation of growth include improved competition for space [70], increased tolerance to fluctuating environmental stressors [71,72] as well as size-based predator avoidance [73]. Reduced growth under environmental stress has been shown in other marine species [74,75]. The delay in early growth that we observed in 6 PSU may be due to altered physiology (e.g. increased osmoregulatory needs), resulting in re-allocation of energy [41] and/or increased shell production costs in low salinities due to low CaCO_3_ content in the water [76].

Salinity had a clear effect on reproduction in all populations with the highest reproductive activity (sum of individuals with fertilized eggs and/or mature gonads) in 15 PSU. Although mature gonads were found in barnacles in 30 PSU, no individuals with fertilized eggs were observed even after nine weeks of study, indicating that reproduction may be delayed in high salinity. This may be a result of more energy initially being invested in shell production in high salinities, revealing a trade-off between two important fitness-related traits: reproduction and defence against predators by producing hard shells (i.e. survival). A similar trade-off between shell morphology and reproduction has also been reported by [77] for the barnacle *Chthamalus anisopoma*. Alternatively, it is possible that reproduction is delayed or disrupted in high salinities, i.e. not representing an adaptive trade-off *per se*, but rather the result of physiological dysfunction. In either case, delaying reproduction will result in fewer broods per year, and thereby affect recruitment dynamics [78,79], however the importance of this delay may be overwhelmed by the effects of season on reproductive activity. Studies on other barnacle species have suggested that exposure to low salinities markedly reduces the reproductive output [80,81]. This does not seem to be the case for *B. improvisus*, providing further support for the conclusion that *B. improvisus* is a truly brackish species.

The calcareous outer shell of a barnacle offers protection against predation but also aids in regulating exposure to extreme conditions such as desiccation or salinity changes [82,83]. The reduction in shell strength we observed at low salinities (Figure 5 and Additional file 1: Table S4) was most likely caused by limited amounts of dissolved CaCO_3_ at low salinity [84,85]. Reduced shell strength and ash weight in response to low salinity has also been found in other invertebrate species, including mussels (*Mytilus edulis*) and oysters [86,87]. Extreme high salinities (>40 PSU) were also found to reduce shell strength of *B. amphitrite*[55], indicating that shell strength may not only be limited by available CaCO_3_ in the water, but may also be associated with other physiological stress in response to changes in salinity.

Interestingly, we found that barnacles from the highest salinity environment (Tjärnö) built stronger shells in 30 PSU compared to the other two populations (Figure 5 and Additional file 1: Table S4). The absence of many of *B. improvisus’* main predators (e.g. *Nucella lapillus*, *Carcinus maenas* and *Asterias rubens*) from lower salinity environments in the inner Baltic Sea [87,88] may have resulted in lower selection pressure for strong shells, especially when CaCO_3_ is limited. Similar, population-specific differences in the ability to build thick shells have been shown for blue mussels (*Mytilus edulis*) from the Baltic and North Seas [88]. Interestingly, barnacles from Kiel (15 PSU) only showed a modest increase in shell strength in 30 PSU, compared to barnacles from Tjärnö (Figure 5 and Additional file 1: Table S4) even though the predators *A. rubens* and *C. maenas* are abundant in that habitat [89]. We can only speculate with regard to the predation pressure that these species exert on *B. improvisus* in Kiel, however it is clear that other factors such as maternal effects [90], trade-offs between shell-strength and reproductive output (CAP analysis, Figure 8), and physiological constraints may also have influenced our results. Furthermore, the influence of gene flow from populations further inside the Baltic Sea (with lower predation pressure) to Kiel is estimated to be higher than the gene flow from Tjärnö to Kiel, based on oceanographic modelling, which could partly explain the observed pattern [91,92].

With the exception of the population-specific responses in shell strength, no evidence of local adaptation was detected in the other measured traits. Several scenarios could explain this result: i) *Balanus improvisus* was first observed in the Baltic Sea relatively recently (<200 years ago [17,93], and this may have been insufficient time for new adaptations to arise – especially if standing genetic variation was low in the founding populations, which is often the case after biological invasions [94-96]; ii) *B. improvisus* has free-swimming pelagic larvae that drift with ocean currents for up to several weeks, potentially creating high levels of gene flow that prevent local adaptation from evolving [97,98]. In addition, *B. improvisus* is a major fouling organism on ships and human mediated dispersal may enhance gene flow between populations [99]. However, there are several examples in the literature that show that adaptations can evolve rapidly [100,101] and that adaptations can be maintained despite high gene flow [25,102,103]; iii) *B. improvisus* displays broad phenotypic plasticity, which can facilitate establishment in new environments without the need for strong selection/adaptation of local populations [104].

Phenotypic plasticity has also been suggested to play an important role in organisms’ ability to cope with current and future climate change [105]. Recent modelling of future climate scenarios in the Baltic Sea indicate that increased precipitation may lead to reduced salinity [23], which could potentially result in dramatic shifts in species’ distributions. According to the most extreme climate scenario of Meier *et al.*[23], the surface salinity of 5 PSU, today situated in the northern Baltic Sea (63° N), would move south to the waters around Bornholm (55° N) by the end of the 21^st^ century (Figure 1) Although hypothetical [3], this type of environmental shift would have dramatic consequences, especially in ecosystems with already low species richness and genetic diversity, such as the Baltic Sea. Our results indicate that it is unlikely that climate-related shifts in salinity will have strong negative effects on *B. improvisus* populations, but may rather favour the species. It should be remembered, however, that climate change involves multiple environmental parameters, as well as shifts in ecosystem composition, and it is therefore hard to predict the likely effects of salinity changes on the ecology of *B. improvisus* or the ecosystems in which it lives.

## Conclusions

In conclusion, we have shown that *B. improvisus* is a highly euryhaline species with strong capacity to tolerate a range of salinities through primarily plastic responses. For almost all response variables, *B. improvisus* performed slightly better at low and intermediate salinities, supporting the idea that *B. improvisus* is one of few truly brackish species. However, the negative effects of low salinity on early initiation of growth as well as reduced shell strength suggest trade-offs between traits in different environments in order to maximise fitness. Population-specific responses in shell-strength indicated the possibility of local adaptation, perhaps in relation to different predation pressures along the salinity gradient. The existence of these population-specific responses, despite recent colonization, high potential dispersal and broad tolerance, supports earlier work indicating that evolutionary changes can occur rapidly. Projected future climate-driven reductions in salinity in the Baltic Sea will most likely not have major impacts on *B. improvisus* populations, however, further work is needed to clarify the interactions between salinity tolerance and other stressors such as temperature, acidification and food limitation [106,107]. Furthermore, selection experiments using multiple generations of *B. improvisus* are needed to elucidate the respective roles of phenotypic plasticity, trans-generational effects, and adaptations in response to locally strong selection pressures. Finally, population genomic studies in the Baltic Sea could help to elucidate population structure and identify (candidate) genes involved in both phenotypic plasticity and local adaptations.

## Competing interests

The author’s declare that they have no competing interests.

## Authors’ contributions

ALW was involved in planning of the experimental design, carried out the experiment and collected the data, ran the statistical analyses and drafted the manuscript. CA participated in designing the experiment, analysing the data and commenting on the manuscript, TL developed the mathematical script for analysis of growth data and commented on the manuscript, UL and AB contributed to the experimental planning and commented on the manuscript, PJ took part in the planning, analyses and commenting on the manuscript, JH was involved in the planning, experimental setup, statistical analysis and commenting on the manuscript. All authors read and approved the final manuscript.

## Supplementary Material

Additional file 1: Table S1 Experimental conditions of the common-garden setup. **Table S2.** Effects of salinity and population on reproduction (frequency of fertilized eggs and mature gonads. **Table S3.** Results of ANOVA testing effects of salinity and population on Gompertz growth model parameters. **Table S4.** Results of ANOVA testing effects of salinity and population on fitness-related traits in barnacles; including ash weight, ash-free dry weight, condition index, shell strength, and mortality). **Table S5.** Results from the PERMANOVA on effects of salinity and population on phenotypic traits in barnacles, including growth, shell strength, condition index and reproductive maturity. Click here for file
