# Supplementary material for: Importance of plasticity and local adaptation for coping with changing salinity in coastal areas: a test case with barnacles in the Baltic Sea
Source: BMC Evol Biol. 2014 Jul 19;14:156. doi: 10.1186/1471-2148-14-156 (PMC4223505; doi:10.1186/1471-2148-14-156)
Supplement: Additional file 1: Table S1 — Experimental conditions of the common-garden setup. Table S2. Effects of salinity and population on reproduction (frequency of fertilized eggs and mature gonads. Table S3. Results of ANOVA testing effects of salinity and population on Gompertz growth model parameters. Table S4. Results of ANOVA testing effects of salinity and population on fitness-related traits in barnacles; including ash weight, ash-free dry weight, condition index, shell strength, and mortality). Table S5. Results from the PERMANOVA on effects of salinity and population on phenotypic traits in barnacles, including growth, shell strength, condition index and reproductive maturity. [file 1471-2148-14-156-S1.pdf]

Additional files

Table S1 - Experimental conditions in the common-garden.

Parameters measured included salinity, temperature, alkalinity and nitrogenous compounds, average ± SD. Water quality was generally assessed just before water exchanges were performed every 14 days. Alkalinity is presented as values just before and just after water exchange, representing minimum and maximum A_T.

Experimental treatment	Salinity	Temperature	Alkalinity	$NH3-NH4^{+}$	NH3
	PSU	°C	umol/L CaCO3	mg/L	mg/L
6 PSU	6.07 ± 0.08	20.20 ± 0.26	706 - 759	< 0.01 - 3.0	< 0.0191
15 PSU	15.05 ± 0.13	20.21 ± 0.24	813 - 1216	< 0.01 - 0.5	< 0.0191
30 PSU	30.16 ± 0.17	20.06 ± 0.31	1189 - 2064	<0.01 - 0.5	< 0.0191

Table S2 - Effects of salinity and population on reproduction.

Effects of salinity and population on the frequency of fertilized eggs (upper table) and frequency of mature gonads but not fertilized eggs (lower table). Significance tests were derived by comparisons of full and reduced models.

**Variable**	**Factor**	**df**	χ^2	**P-value**
Barnacles with fertilized eggs	Salinity	2	28.23	**7.40 x 10^-7**
	Population	2	4.63	0.099
	Sal * Pop	4	5.34	0.254
	Error	35		
Barnacles with mature gonads	Salinity	2	25.01	**3.71 x 10^-6**
	Population	2	12.24	**0.002**
	Sal * Pop	4	1.23	0.874
	Error	35		

Table S3 – Results of ANOVA testing effects of salinity and population on growth.

Results from two-way ANOVA, testing effects of salinity and population on Gompertz growth model parameters (*a*, *b* and *c*).

Source	df	SS	MS	F	P-value	Tukey's post-hoc
GOMERTZ a (shell diameter)						
Salinity	2	1.332	0.666	3.362	**0.0497**	15 > 30
Population	2	0.002	0.001	0.005	0.995	
S x P	4	0.555	0.139	0.670	0.599	
Residual	27	5.350	0.198			
GOMPERTZ b (growth lag phase)						
Salinity	2	8.927	4.463	16.554	**<0.001**	6 > (15, 30)
Population	2	0.742	0.371	1.377	0.270	
S x P	4	0.591	0.148	0.548	0.702	
Residual	27	7.280	0.270			
GOMPERTZ c (max. growth rate)						
Salinity	2	0.028	0.014	1.604	0.220	
Population	2	0.004	0.002	0.231	0.795	
S x P	4	0.035	0.009	1.024	0.413	
Residual	27	0.232	0.009			

Table S4 – Results of ANOVA testing effects of salinity and population on fitness-related traits. Results of two-way ANOVA testing effects of salinity and population on fitness-related traits in barnacles; including ash weight, ash-free dry weight, condition index, shell strength, and mortality).

	Source	df	SS	MS	F-value	P-value	Tukey's post-hoc
(a)	**Ash weight (log)**						
	Salinity	2	94.584	47.292	6.463	**0.005**	6 < (15, 30)
	Population	2	11.962	5.981	0.817	0.452	
	S x P	4	14.846	3.712	0.507	0.731	
	Residual	27	197.577	7.318			
(b)	**Ash-free dry weight**						
	Salinity	2	0.4650	0.2325	1.452	0.252	
	Population	2	0.1350	0.0675	0.422	0.660	
	S x P	4	0.4650	0.1163	0.726	0.582	
	Residual	27	4.3225	0.1601			
(c)	**Condition index**						
	Salinity	2	0.00518	0.00259	8.658	**0.001**	(6, 15) > 30
	Population	2	0.00007	0.00004	0.118	0.889	
	S x P	4	0.00085	0.00021	0.713	0.591	
	Residual	27	0.00808	0.00030			
(d)	**Shell strength**						
	Salinity	2	44418	22209	8.432	**0.001**	
	Population	2	15198	7599	2.885	0.073	
	S x P	4	30301	7575	2.876	**0.042**	
	Residual	27	71119	2634			
(e)	**Mortality**						
	Salinity	2	0.1846	0.0923	5.597	**0.013**	6 < 30
	Population	2	0.0282	0.0141	0.856	0.441	
	S x P	4	0.0348	0.0087	0.528	0.717	
	Residual	18	0.2969	0.0165			

Table S5 – Results of PERMANOVA testing effects of salinity on phenotypic traits in barnacles.

Results from the PERMANOVA on effects of salinity and population on a number of phenotypic traits in barnacles, including growth, shell strength, condition index and reproductive maturity.

Factor	df	SS	MS	Pseudo-F	P (perm)	Permutations
Salinity	2	83.485	41.742	4.9928	**0.001***	998
Population	2	12.572	6.2861	0.75187	0.629	997
SxP	4	28.208	7.0519	0.84347	0.645	999
Res	27	225.74	8.3606			

* Pair-wise test: 6psu ≠ (15psu, 30psu)
